# Validation of deep learning-based fully automated coronary artery calcium scoring using non-ECG-gated chest CT in patients with cancer

**DOI:** 10.3389/fonc.2022.989250

**Published:** 2022-09-20

**Authors:** Joo Hyeok Choi, Min Jae Cha, Iksung Cho, William D. Kim, Yera Ha, Hyewon Choi, Sun Hwa Lee, Seng Chan You, Jee Suk Chang

**Affiliations:** ^1^Department of Radiology, Chung-Ang University Hospital, Chung-Ang University College of Medicine, Seoul, South Korea; ^2^Division of Cardiology, Department of Internal Medicine, Yonsei University College of Medicine, Seoul, South Korea; ^3^Department of Biomedical Systems Informatics, Yonsei University College of Medicine, Seoul, South Korea; ^4^Department of Radiation Oncology, Yonsei Cancer Center, Yonsei University College of Medicine, Seoul, South Korea

**Keywords:** coronary artery calcium score (CACS), chest CT, artificial intelligence, accuracy, cancer patient, risk stratification

## Abstract

This study aimed to demonstrate clinical feasibility of deep learning (DL)-based fully automated coronary artery calcium (CAC) scoring software using non-electrocardiogram (ECG)-gated chest computed tomography (CT) from patients with cancer. Overall, 913 patients with colorectal or gastric cancer who underwent non-contrast-enhanced chest CT between 2013 and 2015 were included. Agatston scores obtained by manual segmentation of CAC on chest CT were used as reference. Reliability of automated CAC score acquisition was evaluated using intraclass correlation coefficients (ICCs). The agreement for cardiovascular disease (CVD) risk stratification was assessed with linearly weighted k statistics. ICCs between the manual and automated CAC scores were 0.992 (95% CI, 0.991 and 0.993, *p*<0.001) for total Agatston scores, 0.863 (95% CI, 0.844 and 0.880, *p*<0.001) for the left main, 0.964 (95% CI, 0.959 and 0.968, *p*<0.001) for the left anterior descending, 0.962 (95% CI, 0.956 and 0.966, *p*<0.001) for the left circumflex, and 0.980 (95% CI, 0.978 and 0.983, *p*<0.001) for the right coronary arteries. The agreement for cardiovascular risk was excellent (k=0.946, *p*<0.001). Current DL-based automated CAC software showed excellent reliability for Agatston score and CVD risk stratification using non-ECG gated CT scans and might allow the identification of high-risk cancer patients for CVD.

## Introduction

The coronary artery calcium (CAC) score has emerged as a useful imaging biomarker for predicting the risk of major cardiovascular events (1). Recent advances in dedicated software that can automatically detect and quantify coronary artery calcification have led to wider clinical application of CAC (2, 3). Traditionally, the CAC score is calculated on electrocardiogram (ECG)-gated CAC-scoring computed tomography (CT); however, studies on lung cancer screening, such as the Dutch-Belgian randomized lung cancer screening trial and the National Lung Screening Trial, have shown a significant association between the CAC assessed on low-dose chest CT and clinical outcome (4, 5). Additionally, recent studies have shown that CAC scores obtained from non-ECG-gated CT are significantly correlated with those obtained from ECG-gated CAC-scoring CT (6–8). These results provide strong evidence for the potential application of CAC scores assessed using non-ECG-gated chest CT in various clinical scenarios.

In particular, CAC assessment could have additional clinical value in patients with cancer. Indeed, the number of cancer survivors is increasing, and cardiovascular disease (CVD) is one of the leading causes of morbidity and mortality in patients with various cancers (9, 10). Most patients with cancer undergo longitudinal CT follow-up for cancer surveillance; however, CAC assessment from non-ECG-gated chest CTs and its clinical application in cancer patients are challenging in real-world practice. As detection of tiny pulmonary metastasis or primary cancer among vast volume of chest CT images is of great importance in patients with cancers, CAC lesions are easily overlooked from non-ECG gated CT scans. We believe that the application of deep learning algorithm-based automated CAC scoring software to non-ECG-gated chest CTs, especially those from patients with cancer, could have a great clinical impact on patient management and prognostication.

Thus, the aim of this study was to validate the recently released deep learning algorithm-based CAC scoring software for non-ECG-gated chest CT scans from patients with cancer and demonstrate its reliability and clinical applicability in a real-world setting.

## Materials and methods

The study was performed in accordance with relevant guidelines and regulations and complied with the Declaration of Helsinki. This study was approved by the institutional review board of Chung-Ang University Hospital. Due to the retrospective nature of the study, the need for patient consent for the use of clinical data was waived by the institutional review board of Chung-Ang University Hospital.

### Study population

**Figure 1:**
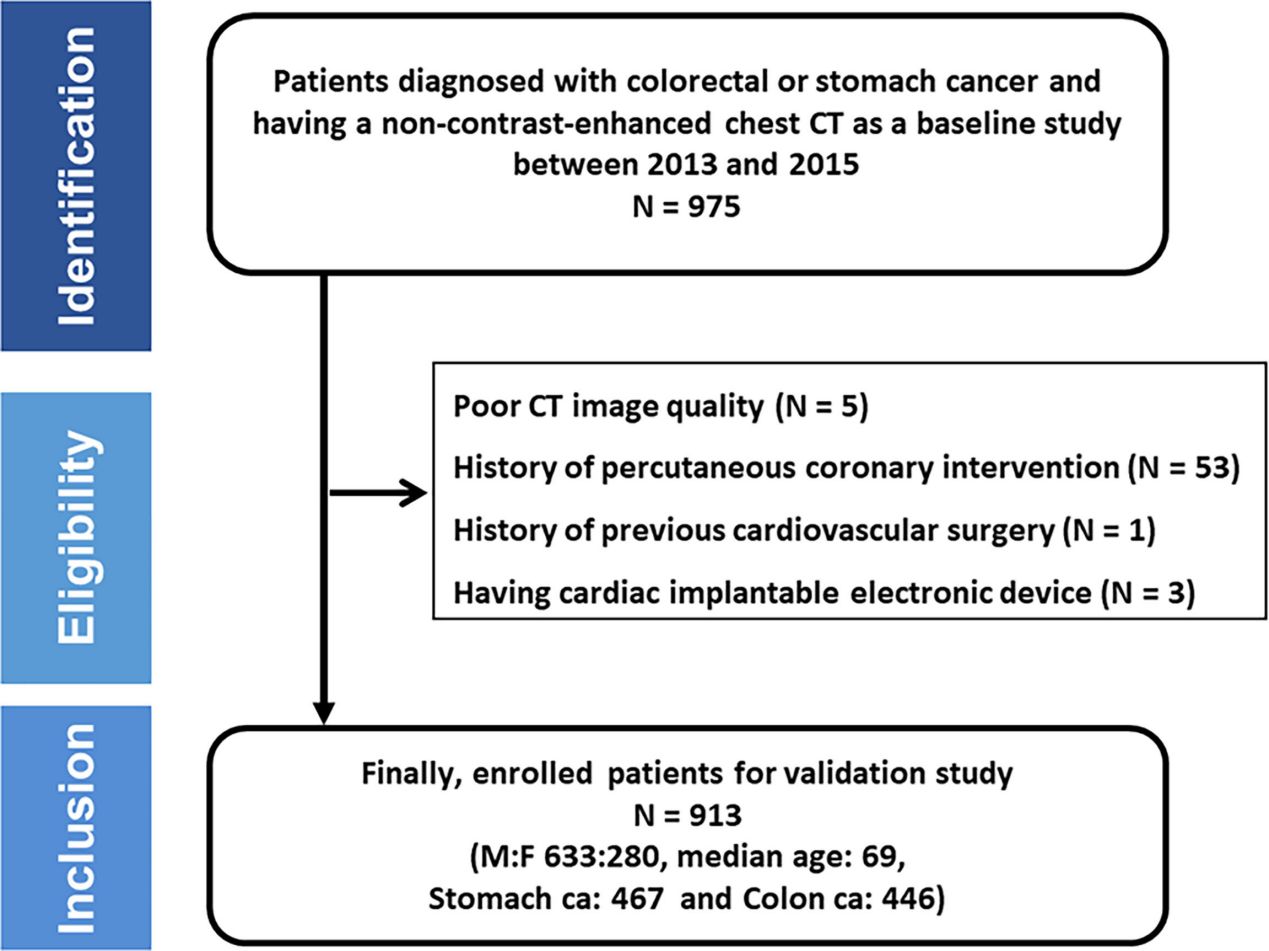
Flow chart of patient enrollment.

We identified 975 patients diagnosed with colorectal or gastric cancer and performed baseline chest CT scans, including non-contrast-enhanced CT images, between 2013 and 2015 at our institution. Of the 975 patients, 61 were excluded due to the following reasons: five for poor image quality; 53 for previous coronary stent insertion; one for previous coronary artery bypass graft; three for having cardiac implantable electronic devices. Finally, 913 patients (633 men and 280 women; median age, 68.3 years) were included in the analysis (**Figure 1**).

### Reference calcium scoring

For reference CAC score determination, non-enhanced chest CT images were analyzed for the presence and extent of CAC according to the Agatston method (11) using dedicated software (Aquarius Workstation, TeraRecon, Inc., San Mateo, CA, USA) by two experienced radiology technologists with at least seven years of experience in CAC scoring. The software overlays CAC lesions having more than 130 Hounsfield units with colors, and the observers manually labeled the CAC lesions according to their anatomical locations (i.e., left main [LM], left anterior descending artery [LAD], left circumflex artery [LCx], and right coronary artery [RCA]) by visually confirming each CAC lesion.

### Automated calcium scoring with software

The automated CAC score was calculated using a commercially available deep-learning-based automatic software (AVIEW CAC, Coreline Soft, Co. Mapo-gu, Seoul, Republic of Korea). The software is an atlas-based CAC acquisition tool, empowered by DL technology (2). For development of the software, the spatial information of coronary and non-coronary regions manually labeled on coronary CT angiography was transfer to non-enhanced CAC scoring CT images using image registration. Then, DL algorithm was developed based on a 3-dimensional U-net architecture for segmentation of coronary and non-coronary regions on CAC scoring CTs (2, 12, 13). Calcium was detected when the potential lesion was in contact with the coronary region, and it did not belong to other structures. The software automatically calculated the CAC score from the uploaded CT images, and the results could be downloaded without the need to open the CT images. In addition, the automatically labeled mask for each CAC lesion was saved by the software.

### Per-lesion comparisons

For cases showing substantial differences in CAC scores (outside the 95% limits of agreement [LOA] on the Bland–Altman analyses), a direct comparison of the measured coronary calcium between the manual and fully automated methods was performed by a cardiovascular radiologist (C.M.J., eight years of experience). The cause and location of each mismatched lesion were analyzed. Then, the mismatched lesions were classified into specific categories, namely, mislabeling of the coronary artery (i.e., LAD calcification labeled as LM), false-positive findings (i.e., aortic wall or valve calcification labeled as CAC), and false-negative findings (i.e., missed CAC on the fully automated method).

### Statistical analyses

The reliability and correlation of the Agatston scores obtained by the fully automated method in comparison with the manual acquisition were evaluated using the intraclass correlation coefficient (ICC), and the Spearman correlation coefficient, respectively. and their agreement was assessed by examining Bland–Altman plots with 95% LOA. Interobserver variability for manual measurements was also assessed using an ICC. An ICC ≤ 0.40 was designated as poor, 0.41–0.60 as moderate, 0.61–0.80 as good, and ≥ 0.81 as excellent agreement (14). In addition, participants were classified into four commonly used CVD risk groups based on Agatston scores as follows: 0 (absent), 0 < CAC ≤ 100 (low), 100 < CAC ≤ 400 (intermediate), and 400 ≤ CAC (high) (15). The reliability of the classification was assessed using the Cohen linearly weighted k statistics, and agreement was assessed using the proportion of participants assigned to the same category by manual and automatic scoring. Values of 0–0.20 were considered as slight, 0.21–0.40 as fair, 0.41–0.60 as moderate, 0.61–0.80 as substantial, and 0.81–1.00 as almost-perfect agreement (16). Statistical analyses were performed using MedCalc software version 19.1.3 (MedCalc Software, Ltd., Ostend, Belgium) and Statistical Package for the Social Sciences version 26 (IBM Corp. Armonk, NY, USA). Statistical significance was set at *p*-value < 0.05.

## Results

### Baseline patient characteristics and CT acquisition parameters

The baseline patient characteristics are summarized in **Table 1**. All CT scans were obtained from patients with colorectal (n=446) or stomach cancer (n=467) for the evaluation of pulmonary metastasis. CT scans were obtained using 256-channel detector CT in 50.6% (462/913), 64-channel detector CT in 38.8% (354/913), and fewer than 64 channels in 10.6% (97/913). All CT images were reconstructed with a soft tissue kernel, and 98.7% (901/913) of the CT scans were obtained at 120 kVp. Regarding slice thickness, 514 of 913 (59.7%) CT scans were reconstructed with a 3-mm slice thickness, 212 of 913 (23.2%) CT scans were reconstructed with a 2.5-mm slice thickness, and 120 of 913 (13.1%) were reconstructed with a 3.75-mm slice thickness.

**Table 1:** Baseline patient characteristics and computed tomography acquisition parameters.

Characteristics	N=913
Age, year	68.2 ± 10.6
Sex (Male: Female)	633:280
Smoking	
Never	650 (71.2%)
Ever	263 (28.8%)
Comorbidities	
Hypertension	283 (31.0%)
Diabetes mellitus	204 (22.3%)
Chronic renal failure	107 (11.7%)
Cardiovascular disease	170 (18.6%)
Cancer type	
Stomach cancer	467 (51.2%)
Colon cancer	446 (48.8%)
CT scanner	
< 64 channel	97 (10.6%)
64 channel	354 (38.8%)
256 channel	462 (50.6%)
Tube voltage	
100 kVp	3 (0.3%)
120 kVp	901 (98.7%)
140 kVp	9 (1.0%)
Kernel	
Soft tissue kernel	913 (100%)
Sharp kernel	0 (0%)
Slice thickness	
< 2.5 mm	3 (0.3%)
2.5 mm	212 (23.2%)
3 mm	545 (59.7%)
3.75 mm	120 (13.1%)
5 mm	33 (3.6%)

### Reliability of the Agatston score measurement

For the Agatston score measurement, the ICC of the total score (per-patient analysis) was 0.992 (95% confidence interval [CI], 0.991 and 0.993, *p*<0.001) between the manual and fully automated methods. On per-vessel analysis, the ICC was 0.863 (95% CI, 0.844 and 0.880, *p*<0001) for the LM, 0.964 (95% CI, 0.959 and 0.968, *p*<0.001) for the LAD, 0.962 (95% CI, 0.956 and 0.966, *p*<0.001) for the LCx, and 0.980 (95% CI, 0.978 and 0.983, *p*<0001) for the RCA (**Table 2**). In terms of correlation, the Spearman’s correlation coefficient was 0.977 (95% CI, 0.974 and 0.980, *p*<0.001) for total Agatston score, 0.736 (95% CI, 0.705 and 0.764, *p*<0.001) for LM, 0.906 (95% CI, 0.893 and 0.917, *p*<0.001) for LAD, 0.897 (95% CI, 0.884 and 0.909, *p*<0.001) for LCx, and 0.935 (95% CI, 0.927 and 0.943, *p*<0.001) for RCA, respectively. When analyzing agreement of scores based on CAC groups, all ICCs were excellent, with 0.912 (95% CI, 0.894–0.926, *p*<0.001) for the absent plus low CAC groups (CAC ≤ 100), 0.939 (95% CI, 0.923–0.952, *p*<0.001) for the intermediate CAC group (100 < CAC ≤ 400), and 0.856 (95% CI, 0.789–0.902, *p*<0.001) for the high CAC group (400 < CAC) (**Supplementary Table S1**).

**Table 2:** Reliability for calculating the Agatston score using deep learning-based fully automated software and manual scoring on chest computed tomography.

	Manual scoring (Ref)*	Automated scoring*	Intraclass correlation coefficient	95% Confidence interval	*p*-value
**Total**	89.8 (15.9 - 272.1)	81.5 (15.4 - 255.3)	0.992	0.991–0.993	<0.001
**LM**	0.0 (0.0 - 21.8)	1.7 (0.0 - 47.0)	0.863	0.844–0.880	<0.001
**LAD**	33.7 (0.0 - 139.0)	20.1 (0.0 - 106.8)	0.964	0.959–0.968	<0.001
**LCx**	0.0 (0.0 - 13.7)	0.0 (0.0 - 10.1)	0.962	0.956–0.966	<0.001
**RCA**	0.0 (0.0 - 50.2)	1.3 (0.0 - 47.8)	0.980	0.978–0.983	<0.001

Per vessel analysis, *median value with interquartile range, LM, left main; LAD, left anterior descending; LCx, left circumflex; RCA, right coronary artery.

**Figure 2:**
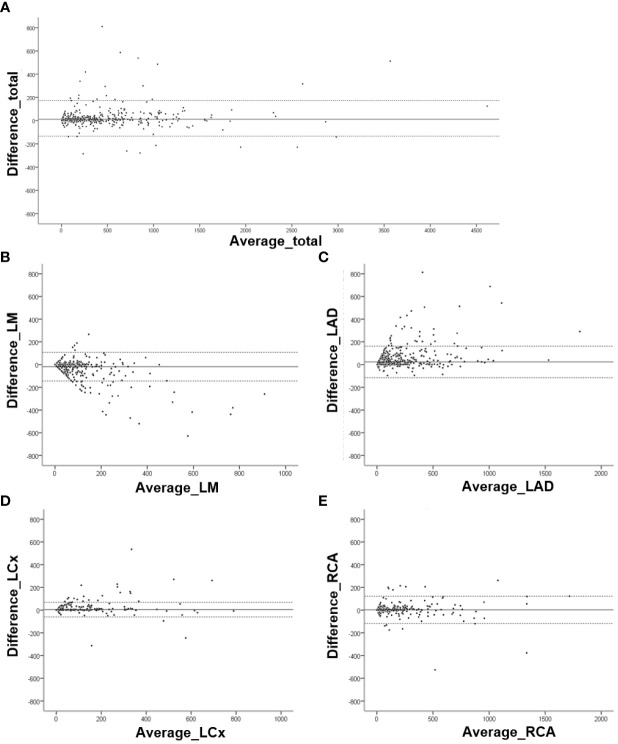
Bland–Altman analysis for Agatston scores obtained by manual and fully automated methods. Graphs for **(A)** Total Agatston score (mean difference, 12.08; 95% limits of agreement [LOA], -133.59 and 157.74), **(B)** left main (mean difference, -18.70; 95% LOA, -145.23 and 107.83), **(C)** left anterior descending (mean difference, 23.56; 95% LOA, -115.14 and 162.27), **(D)** left circumflex (mean difference, 4.55; 95% LOA, -59.88 and 68.97), and **(E)** right coronary (mean difference, 2.09; 95% LOA, 87.73 and 91.91) arteries.

Bland–Altman analyses comparing the CAC scores obtained by the manual and fully automated methods were performed (**Figure 2**). For the total Agatston score, 31 of 913 cases fell outside the 95% LOA. In terms of per-vessel analysis, 42 cases of LM, 33 cases of LAD, 24 cases of LCx, and 24 cases of RCA were outside the 95% LOA.

The inter-reader agreement between the two readers for calculating the manual Agatston scores was excellent (ICC for the total Agatston score, 0.999; 95% CI, 0.999 and 1.000). For per-vessel agreement, the ICC was 0.997 (95% CI, 0.996 and 0.999, *p*<0.001) for LM, 0.999 (95% CI, 0.998 and 1.000, *p*<0.001) for LAD, 0.999 (95% CI, 0.999 and 1.000, p<0.001) for LCx, 0.998 (95% CI, 0.996 and 1.000, p<0.001) for RCA, respectively.

### Risk category assessment

Risk category assessment was analyzed according to CAC-based risk stratification using deep learning-based fully automated software and manual scoring on chest CT. The overall reliability of the CVD risk groups was almost perfect (k=0.946, *p*<0.001) between the manual and fully automated scoring methods (**Table 3**). The majority of patients were assigned to the same CVD risk category (869/913, 95.2%), and 42 patients (4.6%) were assigned to the neighboring risk group.

**Table 3:** Reliability of CAC-based risk stratification using deep learning-based fully automated software and manual scoring on chest computed tomography.

		Deep learning-based fully automated scoring			
		Absent, CAC = 0	Low, 0 < CAC ≤ 100	Intermediate, 100 < CAC ≤ 400	High, 400 < CAC
**Manual scoring (Ref)**	**Absent, ** **CAC = 0**	62	11	0	0
	**Low,** **0 < CAC ≤ 100**	6	395	2	0
	**Intermediate, ** **100 < CAC ≤ 400**	0	13	260	1
	**High, ** **400 < CAC**	0	2	9	152

Kappa, 0.946, 95% Confidence interval = 0.930-0.972, *p* <0.001, CAC, coronary artery calcium. Dark blue cells represent the patients who were assigned to the same CVD risk category, and lighter blue cells represent those who were assigned to the neighboring risk group.

### Per-lesion comparison for the cases out of 95% LOA

**Figure 3:**
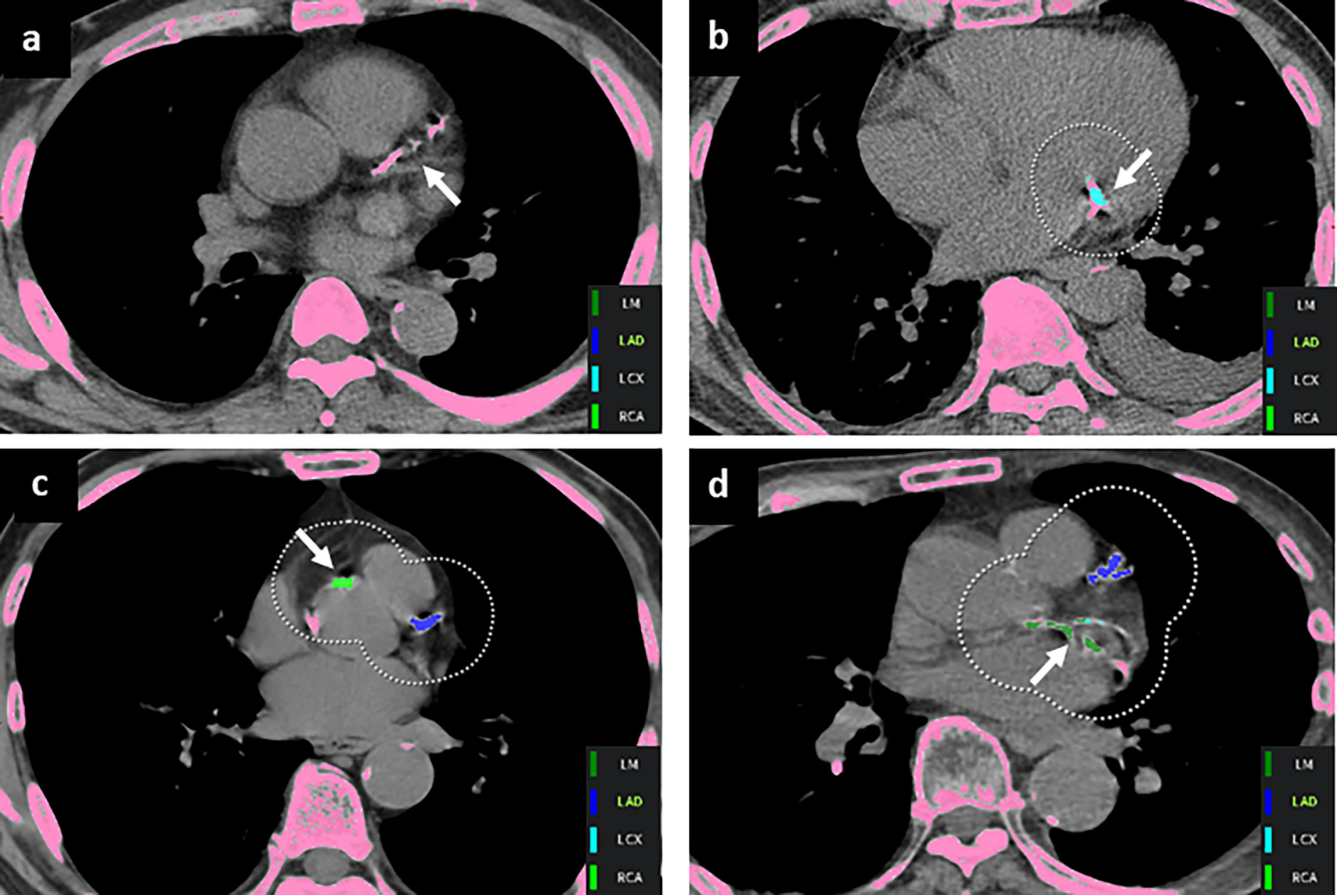
Representative mismatched coronary artery calcium lesions. **(A)** A false-negative finding was noted in the left anterior descending artery (arrow). False-positive lesions were annotated with arrows in the **(B)** mitral annulus and **(C)** aorta wall. **(D)** CAC on the left circumflex artery was mislabeled as the left main artery (arrow).

Per-lesion analysis was performed for cases that were out of the 95% LOA in the Bland–Altman analyses. Overall, 85 cases were reviewed (31 cases for total Agatston score, 42 cases for LM, 33 cases for LAD, 24 cases for LCX, and 24 cases for RCA), and 90 mismatched lesions were identified. Forty percent (36/90) of the mismatched lesions were classified as misnaming of the coronary artery (35 cases of LAD calcification labeled as LM and one case of LCx calcification labeled as LM). Forty out of 90 (44.4%) mismatched lesions were false-negative CACs, which were not detected on automated software, located in the LM (n=2), LAD (n=7), LCx (n=15), and RCA (n=16). False-positive findings included aortic wall or valve calcification (n = 10), mitral annular calcification (n=1), and streaky artifacts with cardiac motion (n=3) (**Table 4** and **Figure 3**).

**Table 4:** Per-lesion analysis for the mismatched CACs in the cases outside of 95% limits of agreement on Bland–Altman analysis.

	Mismatched lesions (n=90)
**Mislabeling of coronary artery**	36 (40%)
**LAD calcification labeled as LM**	35 (38.9%)
**LCx calcification labeled as LM**	1 (1.1%)
**False positive or over-estimation of CAC**	14 (15.6%)
**Image artefacts**	3 (3.3%)
**Aortic wall or valve calcification**	10 (11.1%)
**Mitral annular calcification**	1 (1.1%)
**False-negative or under-estimation of CAC**	40 (44.4%)
**LM**	2 (2.2%)
**LAD**	7 (7.8%)
**LCx**	15 (16.7%)
**RCA**	16 (17.8%)

CAC, coronary artery calcium; LAD, left anterior descending; LM, left main; LCx, left circumflex; RCA, right coronary artery.

## Discussion

Our study demonstrated the clinical feasibility of a fully automated CAC scoring software using non-ECG-gated chest CT scans in patients with gastric and colorectal cancer. Our results showed high reliability for the coronary calcium score measurement and high accuracy for the assignment of risk categories using the fully automated CAC software, currently a commercially available software, compared with conventional manual scoring systems, even with non-ECG-gated chest CTs. The current results can contribute to the wider application of CAC quantification in clinical practice, especially for patients with cancer, as majority of them routinely undergo chest CT for metastasis evaluation.

Previous studies have shown that CAC on non-ECG gated chest CT has prognostic significance (4, 17–19). However, different CAC grading methods were used in many of these studies, leading to a limited general application of the results in routine clinical practice (4, 17–20). Recently, Gal and colleagues showed a strong correlation between an automatically quantified CAC obtained by radiotherapy planning CT and the risk of CVD in patients with breast cancer (Bragatston study) (21).Their study suggested that the CAC score could serve as a surrogate marker for the timely identification of high-risk patients with CVD. To date, CAC has not been fully assessed on chest CT scans due to several factors, including the use of non-ECG-gated CT scans, time-consuming quantification processes, and the lack of generalized grading methods or quantification tools. Our study showed that deep learning-based fully automated CAC scoring software is a fast and accurate tool, potentially enabling the implementation of CVD risk-mitigating strategies in routine clinical practice.

To date, several studies have reported the excellent performance of deep learning models for automated CAC scoring on ECG-gated CT scans (2, 22, 23). Furthermore, several recent studies have demonstrated the application of automated CAC quantification models to non-ECG-gated CT scans, originally used for non-cardiac indications (3, 24). The performance of the deep learning-based automated software used in our study for CAC score acquisition and CVD risk stratification using routine chest CT scans was comparable to or even superior to that of previous studies using deep learning algorithms (3, 24).

Nevertheless, the presence of outliers was still observed. Eighty-five cases outside the 95% LOA on the per vessel analysis revealed 90 mismatched lesions. Forty of these 90 mismatched lesions were CACs that were not depicted as such by the automated software, resulting in false-negative results. In addition, an overestimation of the CAC score resulted from 14 mismatched lesions, which were subsequently found to be calcium deposits in the aortic wall, aortic valve, and mitral annulus. Anatomical errors and misnaming of the coronary arteries were also observed in 36 lesions. Further studies are warranted to eliminate the occurrence of outliers in the deep-learning algorithm.

Our study has several limitations. First, the study was retrospective and conducted at a single center. Second, the CAC was assessed using the Agatston method, and other scales, such as the volume or mass score, were not compared. Third, we did not investigate the cardiovascular outcomes of the participants; thus, the correlation between clinical outcomes and automatically calculated CAC scores could not be evaluated. Finally, we lacked ECG-gated CAC scoring CT as a reference standard. The ground-truth CAC score should be obtained from paired ECG-gated CT; however, previous studies have already shown a strong correlation between the CAC score obtained from non-ECG-gated CT and that obtained from ECG-gated CAC scoring CT (6–8). Thus, as a next step, we focused on the comparison between the manual and fully automatic methods using the same dataset. Further multicenter studies validating the use of automatic CAC scoring software with various CT images and direct comparisons with paired ECG-gated CAC scoring CT should be performed.

In conclusion, we demonstrated excellent reliability for the assessment of CAC scores and CVD risk stratification using deep learning-based fully automated software using non-ECG-gated chest CT scans in patients with cancer. A fast and accurate assessment of CAC can help in the real-world application of CAC quantification on routine chest CTs, which may provide potential opportunities for early prevention in high-risk patients.

## Data availability statement

The original contributions presented in the study are included in the article/**Supplementary Material**. Further inquiries can be directed to the corresponding author.

## Ethics statement

The studies involving human participants were reviewed and approved by the Institutional Review Board of Chung-Ang University Hospital. Written informed consent for participation was not required for this study in accordance with the national legislation and the institutional requirements.

## Author contributions

Conceptualization of the study was suggested by MC, SY and IC. Data curation was done by JHC, MC, YH and HC. Writing the draft manuscript and preparing figures were done by JHC, WDK and MC. All authors reviewed and edited the manuscript.

## Funding

This study was supported by a grant from the National R&D Program for Cancer Control, Ministry of Health & Welfare, Republic of Korea (HA21C0065).

## Conflict of interest

The authors declare that the research was conducted in the absence of any commercial or financial relationships that could be construed as a potential conflict of interest.

## Publisher’s note

All claims expressed in this article are solely those of the authors and do not necessarily represent those of their affiliated organizations, or those of the publisher, the editors and the reviewers. Any product that may be evaluated in this article, or claim that may be made by its manufacturer, is not guaranteed or endorsed by the publisher.
