# Supplementary material for: Validation of deep learning-based fully automated coronary artery calcium scoring using non-ECG-gated chest CT in patients with cancer
Source: Front Oncol. 2022 Sep 20;12:989250. doi: 10.3389/fonc.2022.989250 (PMC9530804; doi:10.3389/fonc.2022.989250)
Supplement: Supplementary file 1 [file Table_1.docx]

**Supplementary Table S1. Subgroup analysis for agreement with the Agatston score according to the CAC score range**

|  | **Manual scoring (Ref)** | **Automated scoring** | **Intraclass correlation coefficient** | **95% Confidence interval** | ***p*-value** |
| --- | --- | --- | --- | --- | --- |
| **Absent + Low, CAC ≤ 100** | 28.4 ± 28.9 | 28.2 ± 32.4 | 0.912 | 0.894–0.926 | <0.001 |
| **Intermediate, 100 < CAC ≤ 400** | 192.7 ± 164.2 | 173.2 ± 175.2 | 0.939 | 0.923–0.952 | <0.001 |
| **High, 400 < CAC** | 605.8 ± 323.0 | 587.8 ± 392.1 | 0.856 | 0.789–0.902 | <0.001 |

Note. CAC = coronary artery calcium
